# Open-label study of treatment with alendronate sodium plus vitamin D in men and women with osteoporosis in Thailand

**DOI:** 10.1186/s12891-018-2309-y

**Published:** 2018-11-06

**Authors:** Thawee Songpatanasilp, Sattaya Rojanasthien, Pansak Sugkraroek, Boonsong Ongphiphadhanakul, Lamar Robert, Chongchit Sripun Robert, Sirichai Luevitoonvechkij, Arthur C. Santora

**Affiliations:** 1 Phramongkutklao Hospital, Bangkok, Thailand; 2 Department of Orthopedics, Faculty of Medicine, Chiang Mai University, Chiang Mai, Thailand; 3 Bumrungrad International Hospital, Bangkok, Thailand; 4 Ramathibodi Hospital, Bangkok, Thailand; 5 Eco-Health-One Health Resource Center, Chiang Mai University, Chiang Mai, Thailand; 6 Merck & Co., Inc., Kenilworth, NJ USA

**Keywords:** Alendronate, Osteoporosis, Thailand, Vitamin D, Beta-CrossLaps (β-CTx), Sunlight exposure

## Abstract

**Background:**

It is generally believed that Thai people do not suffer from hypovitaminosis D because there is abundant sunlight throughout the year, and that taking vitamin D supplements could result in abnormally high levels of vitamin D. This is a Thai FDA-driven study to investigate this risk over a period of 26 weeks of taking alendronate sodium/vitamin D3 combination tablets.

**Methods:**

Osteoporosis patients in Thailand were recruited to a multicenter, open-label, 6-month trial of oral alendronate sodium 70 mg/vitamin D3 5600 IU. Patients received study medication once a week for 26 weeks. Serum 25-hydroxyvitamin D (25(OH)D) and Beta-CrossLaps (β-CTx) levels were measured at baseline and 26 weeks. The primary endpoint was the proportion of patients with 25(OH)D ≥ 50 ng/mL at week 26; it was hypothesized that 26 weeks’ treatment would not result in 25(OH)D serum levels ≥ 50 ng/mL in > 7% of osteoporosis patients.

**Results:**

One hundred ninety-eight patients were recruited. At baseline, 67.2% of the patients had 25(OH)D < 30 ng/mL; this declined to 34.4% by week 26. The mean 25(OH)D level improved from 27.8 ng/mL at baseline to 33.6 ng/mL at week 26. Five patients (2.69% of the full analysis set) had 25(OH)D levels ≥ 50 ng/mL at 26 weeks. The highest 25(OH)D level, 64.3 ng/mL, was observed in a patient whose baseline level was 102.2 ng/mL. The majority (62.9%) of the patients had optimal 25(OH)D levels (30–50 ng/mL). β-CTx levels were reduced by 57.7% after 26 weeks’ treatment. No clinically significant cases of hypercalcemia which could be associated with hypervitaminosis D were identified during physical examination, in vital signs, or in laboratory results. Overall, 73 patients (36.9%) reported at least one adverse event (AE), with 13 (6.6%) reporting drug-related AEs. Four patients discontinued due to AEs, two of which were drug-related. Serious AEs were reported for four patients, of which one was considered drug-related.

**Conclusions:**

Oral alendronate sodium 70 mg plus vitamin D3 5600 IU once weekly had an acceptable safety profile in this study, and increased serum 25(OH)D and reduced β-CTx levels in osteoporosis patients. This treatment improved 25(OH)D levels, without causing abnormally high levels, after 26 weeks’ treatment.

**Trial registration:**

Clinical Trials.gov NCT01437111, Registered September 19, 2011.

## Background

Osteoporosis is characterized by low bone mass and deterioration of bone tissue, which leads to increased bone fragility and risk of fractures [1]. Bone fractures have been shown to increase mortality and morbidity rates among patients with osteoporosis [2]. There is a significant body of evidence indicating that low serum levels of calcium and vitamin D lead to accelerated bone loss [3]. In spite of recommendations to take vitamin D supplements, a high prevalence of vitamin D deficiency has been reported in patients with osteoporosis in various countries [4–6].

Vitamin D is created by photochemical synthesis when skin is exposed to sunlight, and is also obtained from various foods [4, 7]. It has generally been assumed that high levels of exposure to sunlight ensure normal serum vitamin D levels in the Thai population; however, studies have reported conflicting information. One study found no evidence of vitamin D deficiency in healthy elderly Thai volunteers, reporting mean serum 25-hydroxyvitamin D (25(OH)D) levels of 67.4 ng/mL and 42.4 ng/mL in men and women, respectively [8], whereas a subsequent study of healthy elderly Thai women found that 32% had vitamin D insufficiency based on a threshold of 24 ng/mL, although only 0.4% had 25(OH)D levels < 12 ng/mL [9]. The incidence of hypovitaminosis D among older individuals may be due to an age-related decrease in the ability of the skin to synthesize vitamin D [10, 11], or simply to an avoidance of sun exposure at times of the day effective for vitamin D synthesis [11]. Whatever the cause, it appears that vitamin D supplementation is necessary for elderly patients with osteoporosis to achieve adequate vitamin D levels. Many patients with osteoporosis are deficient in vitamin D [12]. The importance of vitamin D in patients with osteoporosis is well established. Optimal vitamin D levels are needed to maximize the effects of antiosteoporotic treatment on bone mineral density (BMD) in patients with osteoporosis [13, 14].

Alendronate sodium is a bisphosphonate that both decreases bone resorption and inhibits osteoclast activity [15–17]. It has been demonstrated to reduce the risk of fracture in patients who have an adequate intake of both calcium and vitamin D [18–20]. Vitamin D is a fat-soluble vitamin and is best absorbed when taken with a fatty meal. The fat provokes the release of bile salts, which in turn facilitate the absorption of dietary fats and fat-soluble vitamins into the lymphatic system and from there into the systemic circulation. By taking vitamin D in a fasted state with only a glass of water, at least half an hour before a meal, the absorption of vitamin D could be much less than if taken with a fatty meal. Although it is convenient to include both vitamin D and alendronate in one tablet, this method and timing of administration would be ideal only for the alendronate and possibly not for the vitamin D. However, a multicenter, international, randomized trial of alendronate 70 mg combined with vitamin D3 5600 IU in a single tablet taken once a week for 6 months showed the benefits of correcting vitamin D insufficiency, increasing BMD, and reducing bone turnover markers in patients who took the combined formulation of alendronate plus vitamin D [21]. A combined formulation of alendronate sodium and vitamin D ensures adequate vitamin D levels to support alendronate efficacy. A combination of alendronate sodium 70 mg/vitamin D3 5600 IU has been approved in Thailand for the treatment of osteoporosis.

Although excessive intake of vitamin D can result in negative health outcomes, primarily hypercalcemia, vitamin D toxicity has not been documented in normal individuals even after prolonged administration of doses up to 10,000 IU per day [22, 23]. Some cases of vitamin D toxicity involving serum 25(OH)D concentrations of > 80 ng/mL and hypercalcemia have been reported after a daily intake of vitamin D > 40,000 IU (1000 μg) [24]. Populations with hypovitaminosis D have been associated with higher all-cause mortality, cardiovascular mortality, and cancers [25–27]. Moreover, epidemiological studies have also shown increased mortality in populations with 25(OH)D levels > 50 ng/mL, with a lower risk of mortality at a 25(OH)D level of 30–49 ng/mL, although this correlation may not be causally related [28].

Thailand, a country in South-East Asia, is located between latitude 6^o^ North and 20^o^ North. There are three seasons in Thailand: hot, rainy, and cool. It is generally believed that due to the amount of sunlight in the tropics, Thai people do not suffer from hypovitaminosis D because there is abundant sunlight throughout the year, and that taking vitamin D supplements could result in abnormally high levels of vitamin D. Due to Thai Food and Drug Administration concerns about this possibility, approval of the combination tablets in Thailand was made conditional, subject to demonstration that the combination of vitamin D 5600 IU once weekly and sunlight exposure does not result in abnormally high serum vitamin D levels in Thai patients with osteoporosis.

Seasonal variation in sun exposure in Thailand is minimal for a combination of reasons. One key factor is that virtually all Thai people actively avoid direct bright sunlight. The level of avoidance increases with the intensity of the sunlight. In the rainy season, the temperature is lower and the cloud cover is greater, so avoidance is reduced. In the cool season, the cloud cover is reduced, but since the cool season in Thailand occurs when the sun is south of the equator, the intensity of sunlight is also lower. In the rainy season, regular cloud cover reduces the intensity of the sunlight. Taken together, these factors result in relatively consistent year-round sun exposure. Groups who are potentially subject to greater sun exposure, such as farmers, construction workers, and others whose employment requires them to be outdoors, regularly take measures to reduce the temperature and, concurrently, to minimize their exposure to direct sunlight. For example, they wear wide-brim hats with an attached scarf that protects the face and neck, as well as long-sleeved shirts. Therefore, this study was conducted over a period of 6 months rather than 12 months due to the strong predilection of the Thai population to actively avoid direct sunlight (especially in the hot season) by staying in the shade and by wearing hats and other protective clothing; this would tend to minimize any potential seasonal bias.

As a result of the potential benefits of vitamin D for patients with osteoporosis [13, 14], the authors deemed it unethical to withhold vitamin D supplementation from patients with osteoporosis who participated in this study. Consequently, this study was designed without a control arm of patients with osteoporosis and no vitamin D supplementation.

The aim of this study was to investigate the effect of alendronate sodium 70 mg/vitamin D3 5600 IU combination tablets for 6 months on 25(OH)D levels in Thai patients with osteoporosis living in Thailand.

## Methods

### Study design

This study was a multicenter, open-label trial of alendronate sodium 70 mg/vitamin D3 5600 IU (Fosamax Plus™ 70/5600, Merck Sharp & Dohme Corp.) in men and postmenopausal women with osteoporosis (NCT01437111).

### Patients

This study was approved by the ethics committee for each of the study sites and was performed in accordance with the ethical standards as laid down in the 1964 Declaration of Helsinki and its later amendments. Patients were recruited from four study sites in Thailand; the planned sample size was 200. Key inclusion criteria were: male aged > 50 years or female who was postmenopausal or had been menopausal for at least 1 year, who met any one of the following BMD criteria: (1) candidate for osteoporosis therapy and BMD T-score ≤ − 2.5 at either the lumbar spine or the total hip or any hip subregion (femoral neck or trochanter); OR (2) candidate for osteoporosis therapy and prior non-pathological fragility fracture (of hip, spine, wrist, humerus, or clavicle) and BMD T-score ≤ − 1.5 at either the lumbar spine or the total hip or any hip subregion (femoral neck or trochanter); OR (3) currently undergoing osteoporosis therapy but meeting one of the two preceding criteria prior to starting osteoporosis therapy plus agreement to discontinue any osteoporosis treatment for the duration of the study (may continue daily multivitamins containing ≤ 400 IU vitamin D). Patients were excluded if they had any unexplained abnormal findings on physical examination or clinical laboratory safety screening tests at baseline.

Patients were classified into three groups with respect to their prior treatment: Group I had received a bisphosphonate within the previous year, strontium within the previous month, and/or estrogen or selective estrogen receptor modulators within the previous 3 months; Group II had received other osteoporosis-related treatments or had received a treatment specified for Group I but outside the specified time window; and Group III were treatment-naïve.

### Treatment

All patients received alendronate sodium 70 mg/vitamin D3 5600 IU combination tablets orally once a week for 26 weeks. Treatment adherence was assessed by counts of returned study tablets.

### Assessments

Fasting serum concentrations of 25(OH)D and Beta-CrossLaps™ (β-CTx) [29] were determined at baseline and at 26 weeks. The half-life of 25(OH)D3 is 15.1 ± 3.1 days [30], thus serum 25(OH)D levels should have achieved steady state after 26 weeks of once-weekly treatment with alendronate 70 mg/vitamin D3 5600 IU.

Safety was assessed by recording adverse events (AEs) at baseline, week 13, and week 26, and during a follow-up telephone call 14 days post-treatment. Safety was also monitored via physical examination and vital signs at baseline, week 13, and week 26 of treatment, and by laboratory tests (blood urea nitrogen, creatinine, calcium, and phosphorus) at baseline and week 26 of treatment.

### Statistical analyses

The full analysis set (FAS) population was included in the analysis of the primary endpoint; this consisted of patients who had received at least one dose of study treatment, had at least one post-baseline observation, and had baseline data for analyses requiring baseline data. A per-protocol (PP) population was included in the analysis of the secondary endpoint. All patients who received at least one dose of study treatment were included in the safety analysis.

The primary hypothesis of the study was that the proportion of patients with serum 25(OH)D ≥ 50 ng/mL would be < 7% at week 26. A secondary endpoint was the percent change from baseline of the bone resorption marker for serum β-CTx at week 26.

Patients were stratified based on their 25(OH)D serum levels into one of four groups: < 20 ng/mL (considered as deficient), < 30 ng/mL (considered as insufficient), ≥ 30 to < 50 ng/mL (considered as optimal), and ≥ 50 ng/mL (considered as high) [4]. Stratification measurements were made at baseline and at 26 weeks.

Descriptive statistics for 25(OH)D at baseline and at 26 weeks were determined for each group of patients as classified by prior treatment. A one-sample z-test was used to determine whether the proportion of patients with 25(OH)D ≥ 50 ng/mL at week 26 was > 7%. A paired t-test was used to compare serum 25(OH)D at baseline and at 26 weeks. Analysis of variance was used to compare serum 25(OH)D between the three groups of patients, as classified by prior treatment. *P*-values < 0.05 were considered statistically significant. All statistical analyses were performed with STATA® software version 10.0 (StataCorp. LLC).

## Results

A total of 198 patients were enrolled in the study, of whom 186 were included in the FAS population and 182 in the PP population. The three patient groups, based on treatment history (Groups I, II, and III), were reasonably well balanced with regard to baseline demographics (Table 1). The observed adherence to the study medication regimen was close to 99.0%.

**Table 1:** Baseline patient characteristics

	Group I (*N* = 29)	Group II (*N* = 70)	Group III (*N* = 99)	Total (*N* = 198)
Gender				
Male, n (%)	2 (6.9)	1 (1.4)	2 (2.0)	5 (2.5)
Female, n (%)	27 (93.1)	69 (98.6)	97 (98.0)	193 (97.5)
Age (years)				
Mean (SD)	66.8 (8.7)	70.1 (8.2)	68.4 (8.4)	68.8 (8.4)
Height (cm)				
Mean (SD)	155.3 (7.2)	151.2 (6.1)	151.4 (6.1)	151.9 (6.4)
Weight (kg)				
Mean (SD)	54.2 (9.0)	51.9 (7.3)	53.3 (8.4)	53.0 (8.2)

Group I: Recent/current bisphosphonate-, strontium-, estrogen-, or SERM-treated patients; Group II: Patients being treated with other drugs; Group III: Treatment-naïve patients. *SERM* selective receptor estrogen modulator

**Fig. 1:**
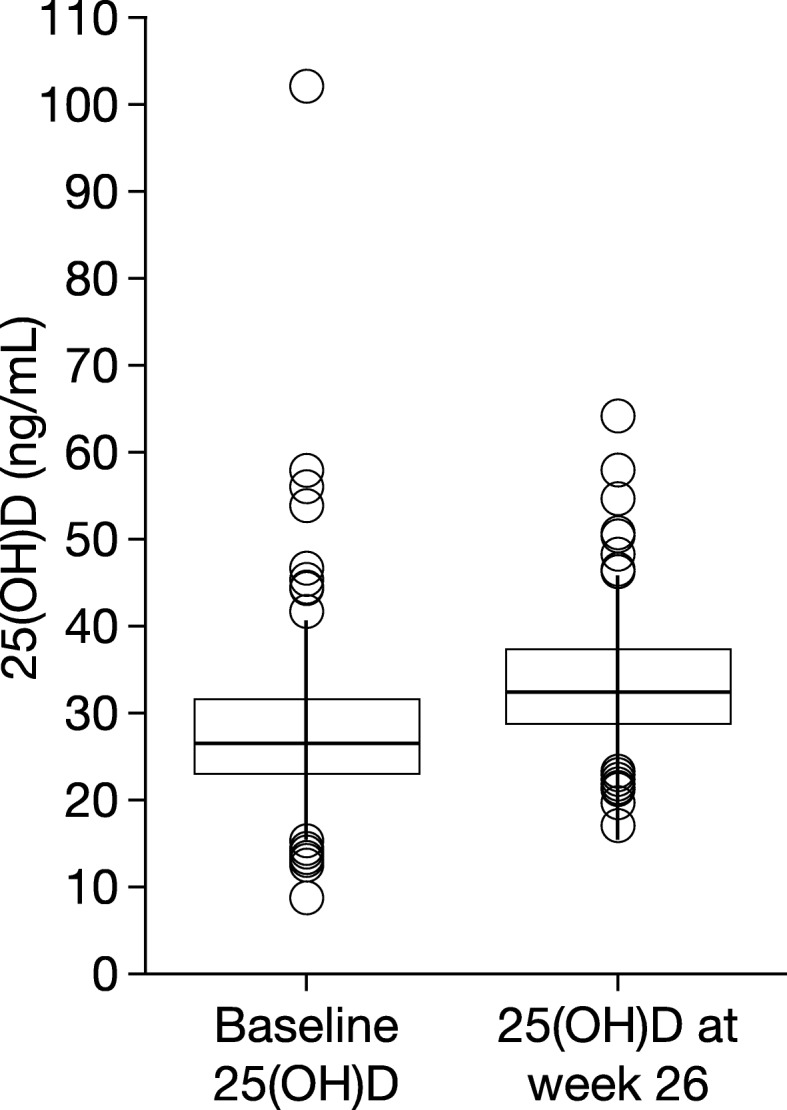
Boxplot of serum 25(OH)D (ng/mL) at baseline and at 26 weeks. 25(OH)D, 25-hydroxyvitamin D

At baseline, 67.2% of the patients had hypovitaminosis D, defined as serum 25(OH)D levels of < 30 ng/mL (Table 2). Treatment with alendronate sodium 70 mg/vitamin D3 5600 IU once a week reduced the proportion of patients with hypovitaminosis D to 34.4% by week 26 (Table 2). In addition, at week 26, mean serum 25(OH)D levels had increased significantly from baseline; for the total patient population, the increase was from 27.8 ng/mL at baseline to 33.6 ng/mL at week 26 (*P* < 0.001) (Table 2 and Fig. 1). Similar increases were seen for each of the three patient groups, and statistically significant changes were seen in Groups II and III (Table 2). The proportion of patients with a 25(OH)D level of ≥ 30 to < 50 ng/mL (defined as optimal) increased from 30.6% at baseline to 62.9% at week 26 (Table 2).

**Table 2:** Serum 25 (OH)D (ng/mL) levels at baseline and at 26 weeks

25(OH)D (ng/mL)	Observation Period	Group I (*N* = 28)	Group II (*N* = 64)	Group III (*N* = 94)	Total (*N* = 186)
≥ 50.0, n (%)	Baseline	1 (3.6)	2 (3.1)	1 (1.1)	4 (2.2)
	26 weeks	1 (3.6)	3 (4.7)	1 (1.1)	5 (2.7)
30.0–49.9, n (%)	Baseline	10 (35.7)	21 (32.8)	26 (27.7)	57 (30.6)
	26 weeks	15 (53.6)	47 (73.4)	55 (58.5)	117 (62.9)
20.0–29.9, n (%)	Baseline	14 (50.0)	36 (56.3)	52 (55.3)	102 (54.8)
	26 weeks	12 (42.9)	14 (21.9)	36 (38.3)	62 (33.4)
< 20, n (%)	Baseline	3 (10.7)	5 (7.8)	15 (16.0)	23 (12.4)
	26 weeks	0 (0)	0 (0)	2 (2.1)	2 (1.1)
Mean (SD)	Baseline	29.7 (8.8)	29.1 (8.4)	26.4 (10.0)	27.8 (9.4)
	26 weeks	33.6 (7.9)	35.3 (7.4)	32.4 (6.8)	33.6 (7.3)
Min, Max	Baseline	16.8, 56.1	14.2, 58.0	8.9, 102.2	8.9, 102.2
	26 weeks	22.7, 50.6	21.9, 58.1	17.2, 64.3	17.2, 64.3
*p* value^a^		0.0913	< 0.0001	< 0.0001	< 0.0001

Group I: Recent/current bisphosphonate-, strontium-, estrogen-, or SERM-treated patients; Group II: Patients being treated with other drugs; Group III: Treatment-naïve patients; ^a^Serum 25(OH)D levels at week 26 compared with baseline. *25(OH)D* 25-hydroxyvitamin D, *SERM* selective receptor estrogen modulator

Only five of 186 patients (2.7%) had 25(OH)D levels ≥ 50 ng/mL at week 26 (*P* < 0.0001). The highest 25(OH)D level, 64.3 ng/mL, was observed in a patient whose baseline level was 102.2 ng/mL. Two of the four patients who had 25(OH)D levels ≥ 50 to < 60 ng/mL at week 26 had baseline levels of ≥ 50 ng/mL. Fewer than 7% of the patients had 25(OH)D ≥ 50 ng/mL after 26 weeks of treatment*.*

The mean baseline β-CTx levels were significantly different in each of the groups in this study (Group I vs. II, *P* = 0.0046; Group I vs. III, *P* < 0.001; Group II vs. III, *P* = 0.0075), with Group 1 showing the lowest level (Table 3). β-CTx levels decreased over the course of treatment in all groups, reaching similar levels after 26 weeks of treatment (Table 3). The mean reduction from baseline in β-CTx for patients in Group I was − 12.7%, which was not statistically significant (*P* = 0.0874), while the change in Group II was − 49.4% (*P* < 0.0001), in Group III was − 76.8%, (*P* < 0.0001), and in the total study population was − 57.7% (*P* < 0.0001) (Table 3). Based on the definition of a reduction in β-CTx of ≥ 30% from baseline constituting a response, 82.3% of patients responded to the treatment, with 73.1% of patients showing a decrease of at least 50% in β-CTx (Table 4).

**Table 3:** Serum bone turnover marker β-CTx (ng/mL) at baseline, at 26 weeks, and changes from baseline

β-CrossLaps (ng/mL)	Group I (*N* = 28)	Group II (*N* = 64)	Group III (*N* = 94)	Total (*N* = 186)
Baseline:				
Mean (SD)	0.162 (0.102)	0.311 (0.205)	0.401 (0.205)	0.334 (0.210)
Baseline:				
Min, Max	0.036, 0.483	0.050, 0.830	0.053, 1.270	0.036, 1.270
26 weeks:				
Mean (SD)	0.121 (0.076)	0.106 (0.058)	0.077 (0.061)	0.094 (0.065)
26 weeks:				
Min, Max	0.032, 0.319	0.032, 0.321	0.017, 0.477	0.017, 0.477
Percent reduction:				
Mean (SD)	− 12.7 (56.9)	− 49.4 (35.5)	− 76.8 (20.2)	− 57.7 (40.3)
Min, Max	− 74.8, 165.8	− 92.8, 34.3	− 96.3, 27.7	− 96.3, 165.8
*p* value^a^	0.0874	< 0.0001	< 0.0001	< 0.001

Group I: Recent/current bisphosphonate-, strontium-, estrogen-, or SERM-treated patients; Group II: Patients being treated with other drugs; Group III: Treatment-naïve patients; ^a^β-CTx levels at week 26 compared with baseline. *25(OH)D* 25-hydroxyvitamin D, *β-CTx* Beta-CrossLaps*, SERM* selective receptor estrogen modulator

**Table 4:** Patients responding to treatment as assessed by serum β-CTx reduction at 26 weeks

Percent Reduction of β-CTx	Group I (*N* = 28)	Group II (*N* = 64)	Group III (*N* = 94)	Total (*N* = 186)
≥ 30%, n (%)	12 (42.9)	44 (68.7)	91 (96.8)	147 (79.0)
≥ 50%, n (%)	7 (25.0)	37 (57.8)	89 (94.7)	133 (71.5)

Group I: Recent/current bisphosphonate-, strontium-, estrogen-, or SERM-treated patients; Group II: Patients being treated with other drugs; Group III: Treatment-naïve patients. *β-CTx* Beta-CrossLaps*, SERM* selective receptor estrogen modulator

There were no clinically significant abnormal findings on physical examination, in vital signs, or in laboratory results (blood urea nitrogen, creatinine, calcium, phosphorus) after 26 weeks of treatment.

A total of 73 patients (36.9%) reported at least one AE; the most commonly reported were upper respiratory tract infections (10 patients [5.6%]), dizziness (9 [4.5%]), and myalgia (7 [3.5%]). Drug-related AEs were reported by 13 patients (6.6%). Four patients (2.0%) discontinued study medication due to AEs; of these, two patients (1.0%) were discontinued due to AEs considered by the investigator to be drug-related (mild myalgia and moderate dyspepsia).

Four patients (2.0%) had serious AEs, with one considered by the investigator to be drug-related. The patient with a serious drug-related AE reported having dyspepsia on the first day of treatment. The patient was admitted to the hospital (the criterion for the “serious” rating) and, after receiving supportive and symptomatic treatment, recovered and was discharged. The investigator rated the AE to be of moderate severity and the patient was discontinued from the study. The other three serious AEs were an intertrochanteric fracture of the left femur (one dose of study medication was missed during hospitalization), hyponatremia thought to be related to concomitant treatment with hydrochlorothiazide (study treatment was discontinued), and tongue cancer (study treatment was discontinued).

## Discussion

There are differing views on optimal vitamin D levels, but hypovitaminosis D is generally considered to be indicated by serum 25(OH)D levels < 30 ng/mL (< 75 nmol/L) [31] and vitamin D deficiency by levels < 20 ng/mL (< 50 nmol/L) [4, 31]. In the current study, approximately two-thirds of the study population had hypovitaminosis D at baseline based on the < 30 ng/mL threshold, but at the end of a 26-week period of once-weekly treatment with alendronate sodium 70 mg/vitamin D3 5600 IU combination tablets, this had been reduced to just over one-third. At the end of the treatment period, 62.9% of patients had 25(OH)D levels in the optimal range (≥ 30 to < 50 ng/mL), compared with 30.6% at baseline. The proportion of patients with serum 25(OH)D levels ≥ 50 ng/mL remained approximately the same throughout the study (2.2% at baseline and 2.7% at week 26). No patient developed signs of vitamin D toxicity during the treatment; the highest 25(OH)D level observed post-treatment was 64.3 ng/mL, but this was lower than the patient’s baseline level of 102.2 ng/mL.

The difference in baseline β-CTx level was in line with expectations based on previous treatment history, with Group I patients (who had been treated with various antiosteoporotic drugs that inhibit osteoclastic activity, thus decreasing bone turnover rate) showing the lowest β-CTx level. The lack of a statistically significant reduction in β-CTx level in Group I can be explained by the low mean baseline values, which are likely the result of previous treatment. Reductions of 49.4%, 76.8%, and 57.7% in β-CTx levels over 26 weeks of treatment were seen in Group II, Group III, and the total study population, respectively. As might be expected, the highest response rate was seen in Group III, which included treatment-naïve patients, with 96.8% of patients showing a decrease in β-CTx from baseline of ≥ 30% and 94.7% showing a decrease of ≥ 50%.

More than one-third of patients in this study reported AEs; however, only AEs reported by 13 patients (6.6%) were considered by the investigator to be drug-related. Four patients (2.0%) discontinued the study due to AEs, two of which were evaluated as drug-related. Serious AEs were reported in four patients. One serious AE considered to be drug-related and resulting in discontinuation from the study was an episode of dyspepsia that led to the patient being admitted to hospital; the investigator rated the event as of moderate severity and the patient recovered fully.

The proportion of patients with hypovitaminosis D at baseline in this study was slightly smaller than that found in a cross-sectional study of 93 elderly Thai women living in institutional long-term nursing homes for the aged, where 77.4% had 25(OH)D < 75 nmol/L (< 30 ng/mL) [32].

Other studies of Thai premenopausal women from five different regions of Thailand [33] and of healthy children in the central region of Thailand [31] also found a high prevalence of hypovitaminosis D (77.8% [using a threshold of ≤ 35 ng/mL] and 79.2%, respectively). One possible reason for the high prevalence of hypovitaminosis D seen across these studies is that, despite high levels of sunlight in Thailand, in the authors’ experience, many people actively seek to minimize their sun exposure.

An optimal 25(OH)D level of ≥ 30 to < 50 ng/mL has been shown to be strongly associated with response to bisphosphonate therapy [17]. Patients with mean serum 25(OH)D levels ≥ 33 ng/mL have been found to have a statistically significantly greater likelihood of maintaining a bisphosphonate response than those with lower levels [15]. This suggests an optimal level of 25(OH)D higher than that recommended by the Institute of Medicine of ≥ 20 ng/mL (≥ 50 nmol/L) [34] may be required to achieve specific therapeutic outcomes.

Short-term changes in biochemical markers have been shown to be valid predictors of long-term changes in BMD in a 6-month study of alendronate treatment in postmenopausal women. In that study, the capacity of change from baseline in β-CTx to predict prevention of bone loss in the spine over 2 years was calculated, with a 50% reduction from baseline β-CTx levels showing sensitivity levels of 78.0%, specificity of 100%, and positive predictive values of 100% [35]. In another study of alendronate treatment in 3105 postmenopausal women receiving treatment for 1 year, greater decreases in biochemical markers were associated with a lower incidence of hip, non-spine, and vertebral fracture [36].

One limitation of the study included the lack of a control arm. The authors deemed it unethical to withhold vitamin D supplementation from the patients with osteoporosis who participated in this study due to the potential benefits of vitamin D in osteoporosis, and because many patients with osteoporosis are deficient in vitamin D [12]. As a result, this research was designed as an open-label study with no control arm; and only limited conclusions can be drawn regarding the influence of treatment on the observed changes in serum 25(OH)D and β-CTx level. It should be noted that similar changes have been observed in other studies of alendronate [15, 34, 35]. Another limitation was the inclusion of patients with abnormally high levels of vitamin D. Furthermore, the study was conducted over a period of 6 months, introducing seasonal variation. However, there is a strong predilection of the Thai population to actively avoid direct sunlight (particularly in the hot season) which could mitigate potential seasonal bias.

## Conclusions

Together with supporting evidence from randomized controlled trials, this study indicates that alendronate sodium 70 mg/vitamin D3 5600 IU taken once a week for 26 weeks is associated with an acceptable safety profile. Furthermore, the study treatment improved the percentage of patients with optimal vitamin D levels, and reduced bone resorption activity in treatment-naïve patients with osteoporosis, patients who had been treated with other drugs, and patients who had not been treated recently for osteoporosis.

## Abbreviations

25(OH)D: 25-hydroxyvitamin D
AE: adverse event
BMD: bone mineral density
FAS: full analysis set
FDA: Food and Drug Administration
PP: per protocol
SD: standard deviation
SERM: selective estrogen receptor modulator
β-CTx: Beta-CrossLaps
